# *In Silico* Design and Analysis of a Novel Ranibizumab-derived Peptide against the Vascular Endothelial Growth Factor

**DOI:** 10.18502/jovr.v21.16222

**Published:** 2026-02-16

**Authors:** Mehrdad Afarid, Roghayyeh Baghban, Samaneh Ghasemali, Javad Zamani, Athar Zareei

**Affiliations:** ^1^Poostchi Ophthalmology Research Center, Department of Ophthalmology, School of Medicine, Shiraz University of Medical Sciences, Shiraz, Iran; ^2^Drug Applied Research Center, Tabriz University of Medical Sciences, Tabriz, Iran; ^3^Bioprocess Engineering Group, Institute of Industrial and Environmental Biotechnology, National Institute of Genetic Engineering and Biotechnology (NIGEB), Tehran, Iran

**Keywords:** Age-related Macular Degeneration, Bioinformatics, Molecular Docking, Molecular Dynamics Simulation, Peptide, Ranibizumab

## Abstract

**Purpose:**

Blocking the interaction between vascular endothelial growth factor (VEGF) and vascular endothelial growth factor receptor-2 (VEGFR2) is recognized as an effective strategy for treating neovascular age-related macular degeneration (nAMD). The present research aimed at designing and modelling an anti-VEGF peptide based on ranibizumab for potential application in inhibiting the VEGF/VEGFR2 interaction.

**Methods:**

Effective amino acids in the interaction between VEGF and ranibizumab were analyzed using Swiss-PdbViewer (SPDBV), PyMOL, and Chimera software. The effective area in this interaction was determined and applied as a basis to design a peptide. Then, this sequence (containing 25 amino acids) was subjected to random mutagenesis, and the binding affinity of the resulting peptides was analyzed using the ClusPro software. Subsequently, GROMACS v5.0.6 was employed for molecular dynamics (MD) simulations to evaluate the stability of target-ligand complexes. Ultimately, the peptide exhibiting the highest affinity was grafted into the kB1 and MCoTI-II frameworks to enhance the stability.

**Results:**

This modification resulted in improved peptide-VEGF binding affinity, demonstrating the potential of *in silico* design for creating effective anti-angiogenic peptides in antiangiogenic therapies.

**Conclusion:**

The findings from this study provide a basis for designing and validating peptide inhibitors against VEGF.

##  INTRODUCTION

Globally, 8.7% of people are affected by age-related macular degeneration (AMD) and lose their vision irreversibly.^[1]^ By 2040, AMD is expected to affect nearly 288 million people worldwide.^[2]^ There are several classifications of AMD based on the degree of vision impairment or the presence of macular neovascularization (MNV). In its early stages, AMD is considered a non-neovascular (dry) condition characterized by macular drusen, either alone or in combination with abnormal retinal pigment epithelium (RPE). In early AMD, vision may not be affected, but late AMD is associated with the loss of central vision. Late AMD manifests in two forms: the “dry” type, characterized by geographic atrophy (GA), and the “wet” type, characterized by MNV.^[3,4]^ Despite accounting for only 10-15% of AMD cases, neovascular AMD causes severe vision loss in most cases.^[5,6]^

Vascular endothelial growth factor A (VEGF-A) contributes significantly to angiogenesis and progression of neovascular AMD. VEGF-A is essential for angiogenesis and progression of neovascular AMD. VEGF-A promotes the growth of vascular endothelial cells and increases vascular permeability. It is specifically crucial for developing embryonic vasculature, including choroidal vasculature. Immunohistological evidence confirms that VEGF is localized in surgically resected choroidal neovascular membranes (CNVs), suggesting that VEGF contributes significantly to their development.^[7,8,9]^ It is thought that VEGF-A is upregulated in neovascular AMD due to complement activation, inflammation, and vascular damage.^[10]^

In recent years, anti-VEGF drugs have revolutionized the field and have been used to treat neovascular AMD. Clinical trials have shown that intravitreal anti-VEGF agents improve best-corrected visual acuity (BCVA) more effectively than photodynamic therapy (PDT) and sham treatment.

Anti-VEGF drugs have also been investigated extensively for the treatment of diabetic macular edema and diabetic retinopathy, and the findings indicate that these diseases regress with these therapeutics.^[11]^

A variety of VEGF inhibitors have been developed for clinical use.^[12]^ The humanized antibody fragment called ranibizumab prevents VEGF molecules from binding their receptors by targeting all VEGF-A isoforms. The Food and Drug Administration (FDA) approved its use in 2006 for neovascular AMD. The MARINA and ANCHOR studies evaluated the effectiveness of ranibizumab for classic neovascular AMD and for minimally classic or occult neovascular AMD.^[13]^ The ANCHOR study compared ranibizumab with PDT over a 2-year period in those with primarily classic subfoveal CNV.^[13,14]^ Eligible patients were older than 50, had a BCVA between 20/40 and 20/320, and had a lesion size < 5400 microns. Three groups were randomly selected from a sample of 423 individuals. Compared to 64.3% in the PDT group, 94.3% of the 0.3 mg group and 96.4% of the 0.5 mg group lost fewer than 15 letters 1 year after starting ranibizumab. Moreover, visual acuity improved by 15 letters or more in 35.7% of the 0.3 mg group and 40.3% of the 0.5 mg group, compared with 5.6% in the PDT group. The improvements persisted after 2 years.^[14]^ Based on visual acuity results, the average improvement from baseline ranged from 8.1 to 10.7 letters, whereas the PDT group showed an average loss of 9.8 letters. In comparison with the control group, ranibizumab also improved the anatomical characteristics of the lesions. The study concluded that ranibizumab, at both doses, was more effective than PDT in treating classic neovascular AMD.^[13]^

A number of promising results have recently been reported for antiangiogenic peptides.^[15,16]^ For ocular diseases, especially retinal diseases, small peptides have advantages over large proteins such as ranibizumab. In therapeutic applications, small peptides are attractive due to their bioavailability, high solubility, and lack of immune reactions. Also, producing small peptides is considerably easier and more controlled than producing proteins.^[17,18,19]^ Numerous strategies have been proposed to enhance the stability and therapeutic efficacy of peptides.^[20]^ However, relatively few peptide-based drugs have successfully reached the pharmaceutical market due to their lower inherent stability compared to small-molecule drugs and their susceptibility to proteolysis. Nevertheless, this limitation can potentially be addressed by employing cyclic disulfide-rich peptides.^[21,22]^ Disulfide-rich macrocyclic peptides are a class of intermediate-sized molecules that hold promise for circumventing certain stability constraints associated with current biopharmaceutical drugs.^[21]^

Naturally occurring cyclic disulfide-rich peptides include SFTI-1 (sunflower trypsin inhibitor-1),^[22]^ MCoTI-II (Momordica cochinchinensis trypsin inhibitor-II),^[23]^ and kB1 (kalata B1).^[24]^ These cyclic peptides exhibit remarkable thermal and enzymatic stability. In the case of MCoTI-II and kB1 peptides, stability stems from the presence of the cyclic cystine knot, whereas for SFTI-1, stability is due to both the cyclic backbone and a robust hydrogen-bonding network.^[25]^ The cyclic disulfide-rich framework loops offer potential for epitope insertion; however, alterations in loop size and structure suggest that certain epitopes may be better suited to certain loops than others.^[26]^

In this study, our objective was to utilize bioinformatics tools to design and model an anti-VEGF peptide. Various bioinformatics software were employed to design this peptide inhibitor. Furthermore, molecular docking analyses were conducted using ClusPro software to evaluate the binding affinity between the selected peptides and the target proteins. Additionally, GROMACS v5.0.6 was utilized for molecular dynamics (MD) simulation to assess the stability of the target-ligand complexes. To increase peptide stability, we employed two disulfide-rich cyclic scaffolds. Specifically, we used two loops from MCoTI-II and six loops from kB1 scaffolds for peptide insertion. The grafting loops in kB1 and MCoTI-II were selected based on previously successful models.

##  METHODS

### Dataset

This study was approved by the local ethics committee of Shiraz University of Medical Sciences, Shiraz, Iran (IR.SUMS.MED.REC.1401.604).

The amino acid sequence of VEGF (entry code: P15692) was obtained from UniProt (http://www.uniprot.org), and the ID for this target protein (1CZ8) was extracted from the Protein Data Bank (http://www.rcsb.org).

### *In Silico* Peptide Design, Molecular Modeling, and Docking

For interactive visualization and molecular structure analysis, we used Swiss-PdbViewer (SPDBV)^[27]^ and LigPlot.^[28]^ These servers provide beneficial data on predicting residues involved in protein interaction sites. The template peptide sequence was achieved based on the VEGF/ranibizumab binding site within an interaction distance of 4 angstroms. Hotspot residues involved in VEGF–ranibizumab binding were identified directly from the crystal structure (PDB ID: 1CZ8) by mapping residues located within 4 Å of the interaction interface using structural analysis tools (SPDBV, PyMOL, Chimera, LigPlot). This structure-based approach ensured that the peptide design was guided by experimentally validated interaction contacts in the antibody–antigen complex.

The AntiCP web server permits the submission of reference peptide sequences of up to 25 amino acids and the design of a single query peptide. All probable mutant analogs of the reference peptide were created, and their anticancer activity and key physicochemical properties, such as charge, pI, and hydrophobicity, were predicted. To predict the structure and function of peptides alone and grafted peptides, we employed the I-TASSER^[29]^ (http://zhang lab.ccmb.med.umich.edu/I-TASSER/) and AlphaFold^[30]^ (https://alphafold.ebi.ac.uk) servers. These servers create 3D *ab initio* modeling and many threading alignments. Likewise, PEP-FOLD3 enables the formation of new conformational alterations after protein-based interaction.^[31]^

Some desirable peptides were modeled by PEP-FOLD. Additionally, the lowest-energy 3D model that demonstrates peptide stability was selected using the PEP-FOLD server. Qualitative model energy analysis (QMEAN)^[32]^ (https://swissmodel.expasy.org/qmean), which computes local and global quality estimates based on single models, may be utilized for assisting model selection and identifying problematic areas that require subsequent refinement.

The binding affinity between virtual peptides and the target protein was evaluated in molecular docking.^[33]^ The optimized PDB structures of peptides were separately subjected to docking using ClusPro 2.0 (https://cluspro.bu.edu/publications.php).^[34]^ As a widely cited docking tool, ClusPro 2.0 was used to predict the peptide-ligand binding orientation. We used the ClusPro server to perform docking studies and examine peptide–receptor interactions using a genetic algorithm. Additionally, the docked solutions were refined using the FireDock web server, and the corresponding dock scores were obtained.^[35]^

### Simulations Setup

MD simulation was applied to refine the structural models derived from the docking study. The structures were refined using GROMACS v5.0.6. The VEGF/peptide complex was placed in a dodecahedron box and then filled with water using the TIP3P water model. To neutralize the systems, some water molecules were replaced with the Cl^–^ or Na+ ions. All systems were analyzed using the NVT and NPT ensembles with periodic boundary conditions. The Parrinello–Rahman algorithm was used to set the pressure at 1 bar under isotropic conditions. Electrostatic interactions were calculated by the PME,^[36]^ and the LINCS^[37]^ algorithm was applied to restrict all bonds between hydrogen atoms.^[38]^ Energy minimization was performed using the steepest-descent algorithm. A 100-ps MD equilibration was performed with positional restraints on protein heavy atoms. This was achieved using a spring constant of 1000 kcal·mol^–1^nm^–2^ in order to prevent nonphysical conformational changes in the solvated protein. The last MD simulation was run for 50 ns without any restraints.

##  RESULTS

### Sequence Availability and Protein Structure Prediction

**Figure 1:**
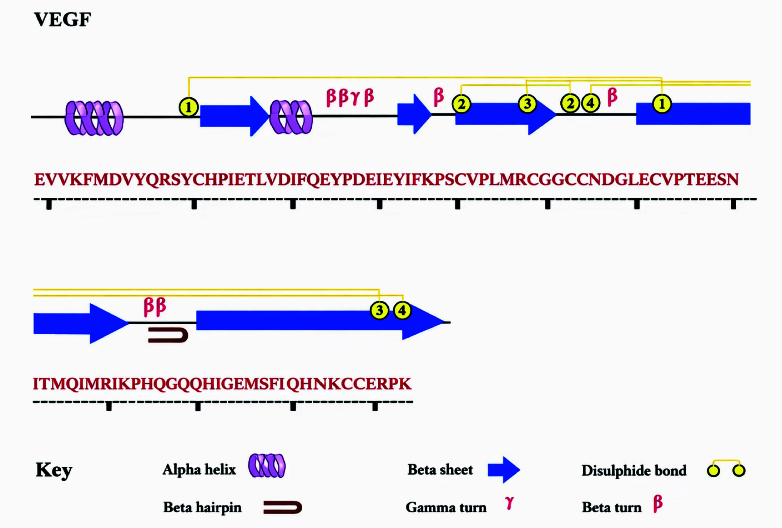
The amino acid sequence of VEGF. This figure illustrates the distribution of secondary structural components and disulfide bonds in the protein structure.

Figure 1 shows the amino acid sequence of VEGF, including α -helices (violet), β -strands (blue), and motifs.

### *In Silico* Peptide Design

**Figure 2:**
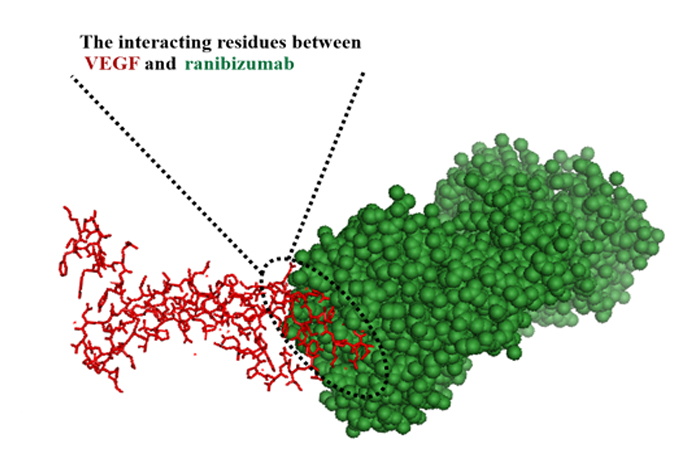
The interacting residues obtained from the VEGF/ranibizumab in 4-angstrom. Red areas in this figure illustrate the YKQIMRIHQGQHIGEM sequence from the VEGF, and the green section is related to the THYGWINTYYPYYYGSW sequence from ranibizumab.

Based on the VEGF/ranibizumab binding site, a template peptide was designed within a 4-angstrom interaction distance. The interaction residues of VEGF and ranibizumab, respectively, were YKQIMRIHQGQHIGEM and THYGWINTYYPYYYGSW. The residues that were selected for designing the peptide were chosen from ranibizumab to neutralize VEGF [Figure 2]. The peptide sequences selected from ranibizumab comprise approximately 25 amino acids, with the residues THYGMNWVRQAPGKGLEWVGWINTY.

### Peptide Modeling and Docking Studies

The AntiCP web server^[39]^ was used to generate some derivatives from the template peptide sequence that led to the development of the “first library” of anticancer peptides. Here, peptides with an SVM score > 0.74 are considered indicative of positive findings. As a result of this procedure, a small peptide library (“second library”) including 16 anticancer peptides was generated [Table 1].

**Table 1:** The peptide sequences analysis using AntiCP

**Name**	**Peptide sequence**	**Mutation position**	**SVM score**	**Mol wt**	**Prediction**
MutA	THYCMNWVRQAPGKGLEWVGWINTY	4	0.76	3010.83	AntiCP
MutB	THYMMNWVRQAPGKGLEWVGWINTY	4	0.76	3038.89	AntiCP
MutC	THYVMNWVRQAPGKGLEWVGWINTY	4		3006.83	AntiCP
MutD	THYGMNWERQAPGKGLEWVGWINTY	8	0.76	2994.73	AntiCP
MutE	THYGMNWVRKAPGKGLEWVGWINTY	10	0.76	2964.79	AntiCP
MutF	THYAMNWVRQAPGKGLEWVGWINTY	4	0.75	2978.77	AntiCP
MutG	THYLMNWVRQAPGKGLEWVGWINTY	4	0.75	3020.86	AntiCP
MutH	THYWMNWVRQAPGKGLEWVGWINTY	4	0.75	3093.91	AntiCP
MutI	THYGMNWCRQAPGKGLEWVGWINTY	8	0.75	2968.75	AntiCP
MutJ	THYGMNWSRQAPGKGLEWVGWINTY	8	0.75	2952.69	AntiCP
MutK	THYGMNWVCQAPGKGLEWVGWINTY	9	0.75	2911.70	AntiCP
MutL	THYGMNWVHQAPGKGLEWVGWINTY	9	0.75	2945.71	AntiCP
MutM	THYGMNWVIQAPGKGLEWVGWINTY	9	0.75	2921.73	AntiCP
MutN	THYGMNWVRCAPGKGLEWVGWINTY	10	0.75	2939.75	AntiCP
MutO	THYGMNWVRIAPGKGLEWVGWINTY	10	0.75	2949.78	AntiCP
MutP	THYGMNWVRTAPGKGLEWVGWINTY	10		2937.72	AntiCP
SVM, support vector machine; Mol wt, molecular weight; AntiCP, anticancer peptide.					

**Figure 3:**
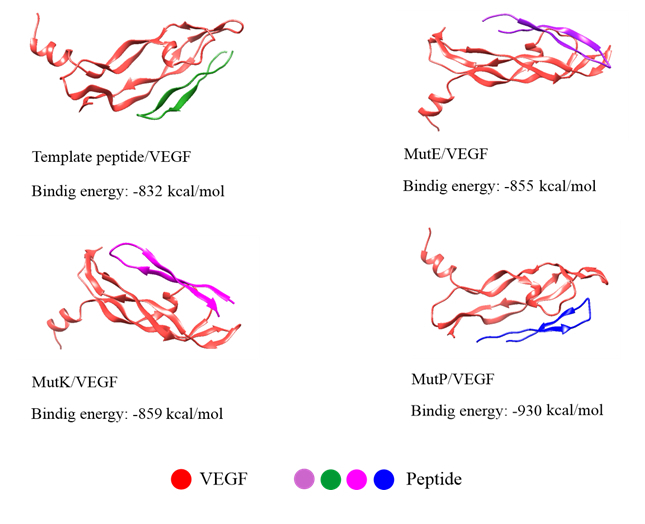
Binding energy and cartoon view of the peptide-VEGF complexes.

Using the I-TASSER server and AlphaFold, the 3D structure of the peptides from the second library was modeled. We observed that the C-score for I-TASSER (0.28) was greater than that of AlphaFold (0.22). Therefore, the results obtained from I-TASSER were used for further analyses. After predicting peptide 3D structures, we assessed the absolute quality of all models using Qmean.^[32]^ After high-quality models were generated, they were docked with VEGF using the online ClusPro server.^[34]^ In this software, the realistic docking modes of protein/protein interactions were directly computed and displayed. Using ClusPro, all 16 peptides of the second library were docked with VEGF. An energy-based scoring function determined all possible binding orientations (translational and rotational) in two molecules of peptides-VEGF. The predicted binding energies of the best docked poses relative to the wild-type peptide are shown in Figure 3.

Three top-scoring peptides are MutE, MutK, and MutP, which correspond to the amino acid sequences of THYGMNWVRKAPGKGLEWVGWINTY, THYGMNWVCQAPGKGLEWVGWINTY, and THYGMNWVRTAPGKGLEWVGWINTY, respectively. Out of the 16 peptides of the second-generation library, three peptides of MutE, MutK, and MutP were selected for simulation due to their high docking scores.

### Conformational Dynamic Stability

**Figure 4:**
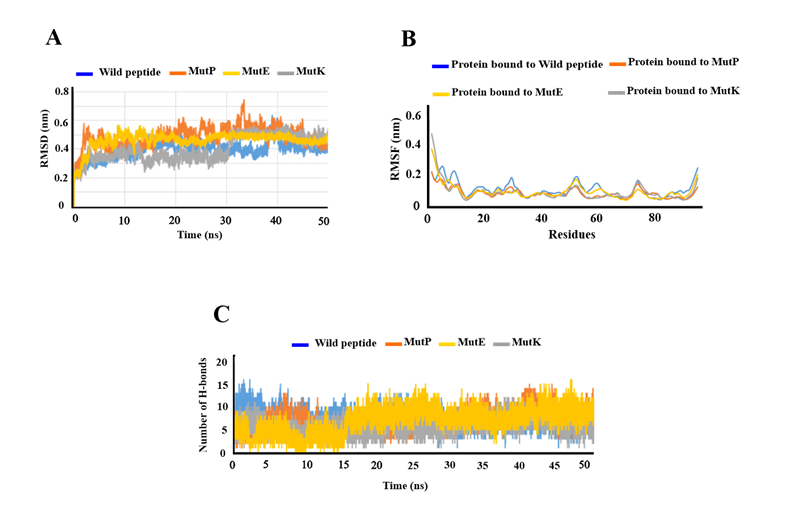
The RMSD, RMSF, and hydrogen bonds of peptide-VEGF complexes. (A) The RMSD plot for peptides has not changed significantly, indicating that the system is in equilibrium. (B) According to the RMSF plot, the highest fluctuation is related to the wild-type peptide. (C) The number of hydrogen bonds per timeframe relative to the average hydrogen-bond count between VEGF and peptides.

The best-docked complexes, refined in FireDock,^[35]^ were subjected to a 50 ns MD simulation to equilibrate interaction areas and acquire an accurate and reliable complex structure close to the truth. These complexes included the wild peptide/VEGF, MutE/VEGF, MutK/VEGF, and MutP/VEGF. Stability analysis was performed by estimating the backbone root-mean-square deviation (RMSD) of the complexes. As a measure of distance between structures, RMSD displays how much the protein structure has transformed throughout simulation [Figure 4]. In the present study, the RMSD values for the protein bound to wild-type and mutated peptides ranged from 0.3 to 0.6 throughout simulation. After 20 ns, the systems reached stability and remained stable thereafter in all complexes, as indicated by the stabilization of RMSDs. RMSF indicates protein residue fluctuations over time from a reference site throughout simulation. RMSF values were determined and are described in Figure 4B.

We did not observe any adverse fluctuations in peptide or protein levels. Residue fluctuations within all complexes were limited to < 0.35 nm, particularly among interior residues, indicating that the interior motif was significantly stabilized by peptide binding. Comparatively, the MutP-bound complex displayed negligible fluctuations at both internal and terminal residues, indicating highly stable interactions.

According to the RMSF results, the wild-type and mutated peptides maintained stable structures of the entire complex, including both internal and terminal residues. In Figure 4C, inter-protein interactions are described according to the number of hydrogen bonds present in the entire trajectory. This plot illustrates the number of hydrogen-bond interactions between two molecules during the simulation.

**Figure 5:**
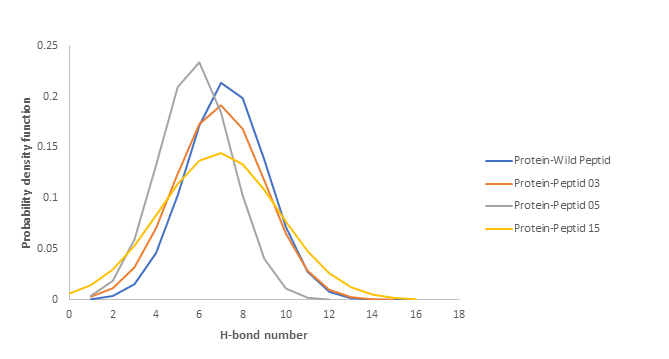
Distribution of hydrogen bonds between proteins and peptides.

A total of 16 high-occupancy inter-molecular hydrogen bonds (H-bonds) were detected between wild-type peptide and VEGF during MD simulations at 1.8 and 2.4 ns. In the MutP/VEGF complex, 15 H-bonds were identified between the two molecules at 42.3, 43.8, and 44.9 ns of the MD simulations. Additionally, 12 H-bonds were observed in the MutK/VEGF complex at 46.8 ns of the MD simulation. In the MutE/VEGF complex, 16 H-bonds were identified at 48.1 and 48.3 ns throughout the simulation. Overall, we observed about seven H-bonds between the protein and all peptides during simulation. Furthermore, the probability distribution of the number of H-bonds in all complexes was plotted. As these plots show, there were 7 H-bonds between the protein and the peptides for most of the simulation time [Figure 5].

**Figure 6:**
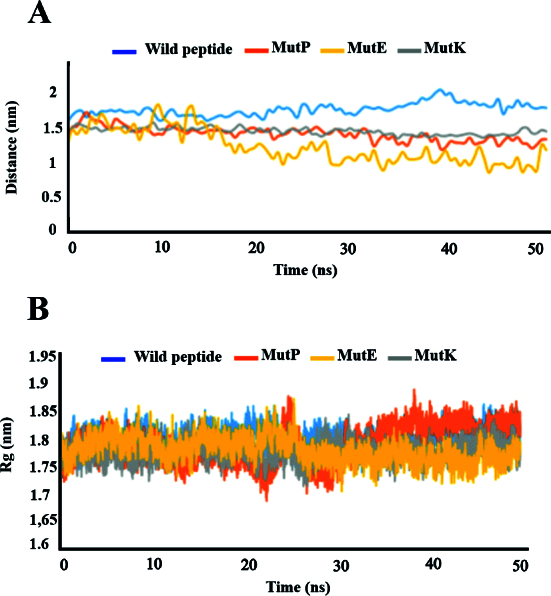
(A) Global center of mass distance between three different peptides and all of the VEGF atoms during the MD simulation. (B) Rg analysis of the compactness of VEGF and peptides during simulation.

The distance between the center of mass of protein and peptides was calculated in three complexes [Figure 6A]. According to the plot, there is no significant change in the distance between the protein and peptide in the three complexes, confirming their structural stability. Radius of gyration (Rg) analysis was performed to assess the compactness of proteins and peptides during simulation; however, we observed no changes in their compactness [Figure 6B].

Furthermore, the SASA (solvent accessible surface area) of the peptide and VEGF complexes was examined to identify any changes in the volume of protein surface. All complexes exhibited a slight upward trend from the beginning, followed by a steady state through the end. Although the SASA trend showed certain deviations, they were minor. The binding free energies between protein and peptide in all complexes were calculated using the molecular mechanics/Poisson-Boltzmann surface area (MM/PBSA) methodology (summary_energy-wild.txt, summary_energy-mut.txt). The results revealed a significant increase in the binding energy of MutP/VEGF compared to other peptides (299.9).

### Molecular Grafting on Cyclic Peptides

**Figure 7:**
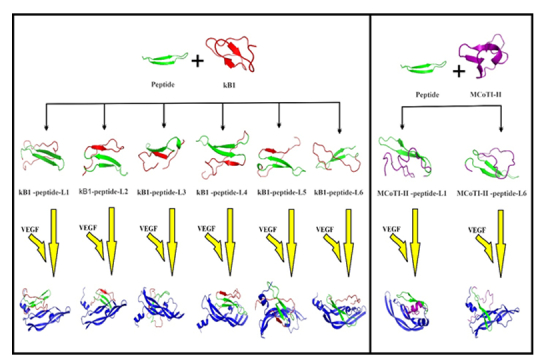
Summary of the grafting procedure for creating anti-angiogenic cyclic peptides, emphasizing the utilization of natural cyclic frameworks (kB1 and MCoTI-II). As depicted, peptides attached to kB1-peptide-L1, kB1-peptide-L3, and MCoTI-II-peptide-L6 do not interact with the target region on VEGF. However, peptides linked to kB1-peptide-L2, kB1-peptide-L4, kB1-peptide-L5, kB1-peptide-L6, and MCoTI-II-peptide-L1 form bonds with the desired region and exhibit favorable binding energies.

The peptide with the highest docking score was chosen and grafted into two loops of MCoTI-II and six loops of the kB1 frameworks [Figure 7]. The figure also illustrates the peptide's three-dimensional structure, both with and without the scaffold. This library of grafted peptides was then subjected to redocking with VEGF, and the resulting docked models for all grafted peptides are presented. Grafted cyclic peptides demonstrating a notably improved global energy were identified and found to have higher affinity than the reference peptide. Upon comparison with the peptide alone and other grafted cyclic peptides, those integrated into loop 4, 5, and 6 of kB1, as well as loop 1 of MCoTI-II, exhibited enhanced peptide-VEGF binding affinity [Table 2].

**Table 2:** The sequences and binding energy of the chosen peptide, the native cyclic peptide frameworks, and the grafted peptides

**Library**	**Sequence**	**Binding energy**
Peptide	THYGMNWVRTAPGKGLEWVGWINTY	–930 kcal/mol
kB1	CGETCVGGTCNTPGCTCSWPVCTRNGLPV	–606 kcal/mol
kB1-peptide-L1	CTHYGMNWVRTAPGKGLEWVGWINTYCVGGTCNTPGCTCSWPVCTRNGLPV	Non-bonded
kB1-peptide-L2	CGETCTHYGMNWVRTAPGKGLEWVGWINTYCNTPGCTCSWPVCTRNGLPV	–914 kcal/mol
kB1-peptide-L3	CGETCVGGTCTHYGMNWVRTAPGKGLEWVGWINTYCTCSWPVCTRNGLPV	Non-bonded
kB1-peptide-L4	CGETCVGGTCNTPGCTHYGMNWVRTAPGKGLEWVGWINTYCSWPVCTRNGLPV	–1049 kcal/mol
kB1-peptide-L5	CGETCVGGTCNTPGCTCTHYGMNWVRTAPGKGLEWVGWINTYCTRNGLPV	–1026 kcal/mol
kB1-peptide-L6	CGETCVGGTCNTPGCTCSWPVCTHYGMNWVRTAPGKGLEWVGWINTY	—1326 kcal/mol
MCoTI-II	CPKILKKCRRDSDCPGACICRGNGYCGSGSDGGV	Non-bonded
MCoTI-II-peptide-L1	CTHYGMNWVRTAPGKGLEWVGWINTYCRRDSDCPGACICRGNGYCGSGSDGGV	–1062 kcal/mol
MCoTI-II-peptide-L6	CPKILKKCRRDSDCPGACICRGTHYGMNWVRTAPGKGLEWVGWINTYGV	Non-bonded

##  DISCUSSION

Angiogenesis contributes to many diseases, including neovascular AMD. Currently available ophthalmic drugs have low bioavailability, high toxicity, and are rapidly degraded by ocular enzymes. In addition, some ocular drugs are less effective due to tachyphylaxis. By identifying crucial angiogenesis regulators, such as VEGF, pigment epithelium-derived growth factor, fibroblast growth factor 2, extracellular matrix molecules, and angiopoietins, it has been possible to develop new therapeutic agents that target the underlying process of pathological angiogenesis. In many forms of ocular neovascularization, VEGF acts as a "master switch" to promote the proliferation and survival of endothelial cells, as well as vascular permeability and ocular inflammation.^[40,41]^ Anti-VEGF agents administered via intravitreal injection are currently the first recommended treatment for AMD.

Despite this approach, some patients continue to experience persistent fluid accumulation or recurring exudation, suggesting a potential resistance to anti-VEGF therapy. However, there is no universally accepted definition of what constitutes anti-VEGF resistance.^[42]^ Tranos et al proposed that resistance may be observed in patients who do not improve or respond to current therapies.^[43]^ Bakall et al presented a broader perspective, noting that resistance can manifest in two ways: either as an initial weakness or lack of response to treatment, or as a gradual decline in treatment efficacy after an initial successful outcome.^[44]^ This lack of agreement on the definition of anti-VEGF resistance underscores the complexity of AMD treatment and the need for further research to better understand and address treatment-resistant cases. Since angiogenesis is largely regulated by VEGF, disrupting VEGF–VEGFR complexes could be an effective treatment.^[45]^

Ranibizumab is a humanized antigen-binding fragment (Fab) that neutralizes VEGF-A isoforms and their biologically active degradation products.^[46]^ Ranibizumab, the first FDA-approved treatment for neovascular AMD, is effective in approximately 25 to 30% of patients with CNV subtypes. In these treated patients, vision is maintained or improves in ≥ 90% of cases, and visual acuity improves by ≥ 15 letters.^[47]^

In the development of antiangiogenic medications, designing peptides that inhibit angiogenesis is a major focus of research.^[26]^ Some anti-VEGF peptides have been identified through rational design, library screening, and phage display.^[48]^ Using this approach, an antagonist peptide of VEGF-A and VEGF-B was generated by covalently linking two selected sporadic receptor-binding regions of VEGF-B to a receptor-binding region of VEGF-A. Furthermore, the previously developed peptide, VGB4, can bind to both VEGFR1 and VEGFR2.^[49]^ In the current study, docking and MD simulation studies were performed to computationally evaluate several wild-type and mutant peptides against VEGF. Accordingly, several peptides were designed computationally using a variety of approaches. To design the peptides, we selected interface residues involved in the interaction between VEGF and ranibizumab. The anti-CP server was used to obtain mutant peptides, starting with the THYGMNWVRQAPGKGLEWVGWINTY sequence. We also used bioactivity and anticancer properties to validate all the peptides in order to produce a peptide capable of functional activity. In comparison with the wild-type peptide, docking of the mutant peptides resulted in a much higher dock score. This indicates that mutation or modification of the wild-type peptide raises its binding affinity.

The best-docked complexes, including the wild peptide/VEGF complex, MutE/VEGF complex, MutK/VEGF complex, and MutP/VEGF complex, were assessed for stability. All complexes were found to be stable. In all complexes, a minimum RMSD deviation showed that peptide binding stabilizes the conformation of VEGF. Moreover, it was noted that residue fluctuations of the MutP/VEGF complex were less considerable than those of the other complexes, suggesting stable residue conformation within this structure. Interprotein interactions revealed that all peptide-docked complexes had a comparable number of hydrogen bonds, confirming the stability of these complexes. Besides, dock scores obtained from FireDock showed no correlation with the predicted hydrogen bonds along the trajectory. The inconsistency between trajectory analysis and dock scores was explained by the scores derived from the static models of the VEGF-peptide complex. The best (lowest) global energy was related to the MutP peptide, with the sequence THYGMNWVRTAPGKGLEWVGWINTY, exhibiting the best-docked solution. As a result, this peptide is considered the most optimal in terms of binding energy. Therefore, the MM-PBSA method was used to estimate hydrogen bond stability and persistence throughout the simulation. Comparing the peptide alone with those grafted on cyclic peptides, it was discovered that the peptide grafted onto loop 4, 5, and 6 of kB1, as well as loop 1 of MCoTI-II, demonstrated a higher binding affinity for VEGF.

In summary, the findings of this study highlight the potential of *in silico* design to identify novel and potent anti-angiogenic peptides and, thereby, to develop anti-angiogenic therapies. Among the variants analyzed, MutP was selected as the most favorable mutant variant due to its best global energy score of 299.9. The peptide sequence THYGMNWVRTAPGKGLEWVGWINTY exhibited the best docking results, indicating its optimal binding characteristics. MutP was considered the optimal peptide based on binding energy, a critical factor in our analysis. Additionally, the RMSF values provided valuable insights into the flexibility and dynamics of individual atoms within the molecular system. The residue fluctuations observed in the MutP/VEGF complex were lower than those observed in other complexes, suggesting a more stable residue conformation. Among the peptides studied, MutP exhibited greater compactness and stability throughout the simulation process. Furthermore, this variant exhibited acceptable results in other analyses as well. Overall, the MutP/peptide complex exhibited more stable interactions than the other studied variants.

##  Financial Support and Sponsorship

None.

##  Conflicts of Interest

None.
